# Gut Microbiota, Muscle Mass and Function in Aging: A Focus on Physical Frailty and Sarcopenia

**DOI:** 10.3390/nu11071633

**Published:** 2019-07-17

**Authors:** Andrea Ticinesi, Antonio Nouvenne, Nicoletta Cerundolo, Pamela Catania, Beatrice Prati, Claudio Tana, Tiziana Meschi

**Affiliations:** 1 Geriatric-Rehabilitation Department, Azienda Ospedaliero-Universitaria di Parma, Via Antonio Gramsci 14, 43126 Parma, Italy; 2 Microbiome Research Hub, University of Parma, Parco Area delle Scienze 11/A, 43124 Parma, Italy; 3 Department of Medicine and Surgery, University of Parma, Via Antonio Gramsci 14, 43126 Parma, Italy

**Keywords:** gut-muscle axis, probiotics, geriatrics, grip strength, gait speed, disability

## Abstract

Human gut microbiota is able to influence the host physiology by regulating multiple processes, including nutrient absorption, inflammation, oxidative stress, immune function, and anabolic balance. Aging is associated with reduced microbiota biodiversity, increased inter-individual variability, and over-representation of pathobionts, and these phenomena may have great relevance for skeletal muscle mass and function. For this reason, the presence of a gut-muscle axis regulating the onset and progression of age-related physical frailty and sarcopenia has been recently hypothesized. In this narrative review, we summarize the studies supporting a possible association between gut microbiota-related parameters with measures of muscle mass, muscle function, and physical performance in animal models and humans. Reduced muscle mass has been associated with distinct microbiota composition and reduced fermentative capacity in mice, and the administration of probiotics or butyrate to mouse models of muscle wasting has been associated with improved muscle mass. However, no studies have targeted the human microbiome associated with sarcopenia. Limited evidence from human studies shows an association between microbiota composition, involving key taxa such as *Faecalibacterium* and *Bifidobacterium*, and grip strength. Similarly, few studies conducted on patients with parkinsonism showed a trend towards a different microbiota composition in those with reduced gait speed. No studies have assessed the association of fecal microbiota with other measures of physical performance. However, several studies, mainly with a cross-sectional design, suggest an association between microbiota composition and frailty, mostly assessed according to the deficit accumulation model. Namely, frailty was associated with reduced microbiota biodiversity, and lower representation of butyrate-producing bacteria. Therefore, we conclude that the causal link between microbiota and physical fitness is still uncertain due to the lack of targeted studies and the influence of a large number of covariates, including diet, exercise, multimorbidity, and polypharmacy, on both microbiota composition and physical function in older age. However, the relationship between gut microbiota and physical function remains a very promising area of research for the future.

## 1. Introduction

### 1.1. Sarcopenia and Physical Frailty

Sarcopenia is an age-related generalized skeletal muscle disorder characterized by loss of muscle mass and a reduction of muscle function, increasing the risk of negative outcomes such as falls, fractures, disability and mortality [1,2]. This condition frequently overlaps with physical frailty [3], representing the reduction of the capacity to maintain fitness, physical performance and sense of well-being over time and particularly after stressors such as acute illnesses [4]. In fact, frail older patients frequently experience a decline in muscle strength, gait speed and endurance capacity [5,6], ultimately leading to the loss of independence in daily activities, increased risk of falls, fractures, hospital admissions, and death [7]. Although sarcopenia and physical frailty remain two distinct conditions with different diagnostic criteria, they share a common pathophysiological background and, from the patient’s perspective, are associated with similar negative outcomes [3,8]. Therefore, they are increasingly viewed as two sides of the same coin. 

The age-related mechanisms promoting the onset of sarcopenia and physical frailty include inflammation, immunosenescence, anabolic resistance and increased oxidative stress [9,10]. These mechanisms are enhanced in case of sedentary behavior and protein-energy malnutrition, either due to physiological age-related loss of appetite called “anorexia of aging” or disease-related increases in energy needs [11,12,13]. Thus, exercise and nutritional supplementation are considered the current pillars for the treatment and prevention of physical frailty and sarcopenia [14,15]. Anabolic hormones could also have a possible therapeutical role in selected situations, where hormonal deficiency can be demonstrated by clinical examinations [16]. 

In some patients, sarcopenia and physical frailty may coexist with reduced bone mineral density (BMD) and excess adipose tissue, in a clinical picture that has been recently defined as osteosarcopenic obesity [17,18]. One of the main features of this syndrome is the presence of adipose tissue and adipose cells within the muscle structure, the so-called “myosteatosis” [17]. Myosteatosis represents the result of increased systemic anabolic resistance and is independently associated with increased mortality risk according to population-based studies [19,20]. 

### 1.2. Gut Microbiota: A Novel Player in Sarcopenia and Physical Frailty?

In this scenario, researchers are increasingly focusing their interests on the possible involvement of gut microbiota in the pathophysiology of physical frailty and sarcopenia. Alterations in the gut microbiota composition could in fact promote chronic inflammation and anabolic resistance, ultimately conditioning reduced muscle size, impaired muscle function and adverse clinical outcomes [21,22]. In this possible gut-muscle axis, age-associated dysfunction of the gut mucosal barrier function may play a central role [23], favoring the entry of microbial products or microbes themselves into systemic circulation [24], and contributing to activate the inflammatory response and induce immune system dysregulation [25,26]. 

Experimental models of aging have shown that the age-associated modifications in gut microbiota composition promote intestinal mucosa permeability. This phenomenon results into increased systemic absorption of bacterial products, including lipopolysaccharide, activating the inflammatory response and ultimately resulting into increased circulating levels of pro-inflammatory cytokines, such as interleukin-6 and tumor necrosis factor-α [27]. Although these mechanisms have not been confirmed in humans yet, the relevance of systemic inflammation for the pathophysiology of sarcopenia has been demonstrated by many investigations [28].

Additional possible mechanisms include microbiota-related modifications in bioavailability of nutrients and production of mediators exerting favorable metabolic activity on the host, such as short-chain fatty acids (SCFAs) [22], or toxins exerting negative effects, such as indoxyl sulfate [29]. Namely, the circulating levels of microbiota-derived indoxyl sulfate are positively associated with the expression of myostatin and atrogin-1 [29], representing two of the main negative regulators of skeletal muscle mass [30]. 

The microbiota can also modulate the “anorexia of aging” phenomenon. In fact, microbial metabolites may act as endocrine modulators of appetite and influence the enteric nervous system signaling to the brain [31,32]. In experimental models, the abundance of key taxa associated with modulation of inflammation, such as *Escherichia coli*, is positively correlated with satiety perception and with satiety hormone levels [32]. These findings imply that, in older patients, the microbiome could influence the onset of sarcopenia and physical frailty also by promotion of malnutrition. 

Gut microbiota is also able to regulate fat storage in many organs [33], and obesity has been associated with a distinct gut microbiota composition, as compared with lean phenotype [34]. The aging microbiota could thus be pathophysiologically involved in the onset of osteosarcopenic obesity. Microbiota-derived SCFAs exert profound influences on skeletal muscle cell function by promoting mitochondrial activity [35]. An optimal mitochondrial fatty acid oxidation in the muscle is fundamental for skeletal muscle remodeling and for limiting myosteatosis [36,37]. Reduced SCFA production by the aging microbiota could thus promote insulin resistance, reduce mitochondrial fatty acid oxidation, and result into an increased intramuscular fatty acid deposition. This phenomenon leads to reduced muscle strength and quality, and further promotes insulin resistance, favoring the onset of a vicious cycle that ultimately leads to sarcopenia and physical frailty [38,39]. 

Over the age of 70, the gut microbiota composition and functionality face complex changes that generally do not occur during adult life [40]. Increased inter-individual variability of composition, reduced biodiversity, and overgrowth of pathobionts are the main features of the elder microbiota [41,42], and meet the definition of dysbiosis, i.e., every change in gut microbiota composition and functionality that implies significant derangements for the host physiology and increased risk of infection [40]. This condition has been associated with several acute and chronic illnesses, involving not only the gastrointestinal system, and is considered able to influence the pathophysiology of several organs [40,42,43]. However, a causal relationship between dysbiosis and human diseases has been demonstrated in only few cases [43]. 

The microbiota composition is in fact deeply influenced by environmental factors, including diet, lifestyle, and medication use [43,44]. In older individuals, these factors may contribute to shape the microbiota composition, so that dysbiosis may represent a consequence, rather than a cause, of a poor health status [44]. Healthy, active older subjects, including centenarians, show a microbiota composition resembling that of adult subjects, with limited features of dysbiosis [45,46]. Conversely, disabled nursing home residents generally exhibit pronounced dysbiosis in fecal samples [47]. 

In sarcopenia and physical frailty, the intestinal microbiota may thus represent a cross-road mediator, transducing environmental stimuli into physiological processes, rather than an etiologic factor [22]. Unfortunately, the current literature state-of-the-art does not allow to disentangle this dilemma, since, despite a high interest into age-related microbiome changes, studies specifically focusing on sarcopenia are still lacking [48]. However, a number of studies, performed on both animal models and human beings, have investigated the possible association of gut microbiota-related parameters with measures of muscle mass, muscle function and physical performance. 

The aim of this narrative review is to critically summarize the possible association of gut microbiota-related parameters with measures of muscle mass, muscle function and physical performance. 

Papers were searched for on PubMed as of 1 June 2019, using the following key terms: microbiota AND “muscle function”; microbiota AND sarcopenia; microbiota AND “muscle mass”; microbiota AND “muscle wasting”; microbiota AND frailty; microbiota AND “gait speed”; microbiota AND “muscle strength”; microbiota AND “grip strength”; microbiota AND “physical performance”. Due to the low number of human studies performed on the topic, and to heterogeneity of settings, participants and outcome measures, a systematic review approach was not possible. Thus, human studies relevant for the primary aim of the review were discussed, considering also animal studies relevant for the explanation of possible pathophysiological mechanisms involved in the gut-muscle axis. 

## 2. Gut Microbiota and Muscle Mass

### 2.1. The Microbiota as Transducer of Nutrient Signals

Studies performed on mouse models have shown that the intestinal microbiota consistently influences the host metabolic balance. Germ-free mice exhibit a persistently lean phenotype even when fed a high-fat diet [49]. Transplantation of fecal microbiota from undernourished humans to germ-free mice also resulted in growth deficits even in the presence of a balanced diet [50]. Finally, transplantation of the intestinal microbiota from pigs to lean germ-free mice resulted in significant changes of muscle fibers structure, resembling to that typical of pigs [51]. Thus, the microbiota may act as a fundamental transducer of nutrient signals to the host and, in physiological situations, diet itself contributes to shape microbiota composition and functionality [52]. 

Protein intake has a recognized pro-anabolic effect on skeletal muscle, favoring deposition of muscle mass in synergy with physical exercise [53,54]. This effect may be mediated by the gut microbiota. In broiler chickens, the rate of muscle mass growth under similar dietary regimens is deeply influenced by specific gut microbiota metabotypes, suggesting a fundamental role of gut microbiota for amino acid absorption and promotion of muscle anabolism [55]. In a recent randomized controlled trial performed on 38 overweight human beings, receiving a three-week isocaloric supplement containing casein and soy protein or maltodextrin as control, the protein supplementation resulted in a significant shift of bacterial metabolism towards amino acid degradation and fermentation [56]. This means that the intestinal microbiota can contribute to promote protein anabolism in the host, by increasing amino acid bioavailability and stimulating insulin secretion and responsiveness in the skeletal muscle. In fact, animal studies have demonstrated that an increased production of branched-chain amino acids (BCAAs) by the microbiota, typical of a normalized Firmicutes/Bacteroidetes ratio, is associated with improved insulin sensitivity and protein synthesis [57]. However, these mechanisms have been put into question by studies conducted in human beings, where elevated serum levels of BCAA are generally associated with insulin resistance [57]. 

The effects of high-protein diets on the microbiota may in fact not always be favorable for the muscle. In mice fed with high-protein diets, the gut microbiota generally faces reduction of Firmicutes/Bacteroidetes ratio and increases in the representation of pathobionts, such as Enterobacteriaceae, at the expense of taxa producing metabolic modulators such as SCFAs, with weight loss and possible negative consequences for muscle metabolism, including reduced modulation of inflammation and increased insulin resistance [58,59,60,61]. Similar results have been obtained by a human randomized controlled trial where the long-term administration of a beef protein supplement to endurance athletes resulted in a reduced fecal microbiota representation of health-related taxa, including *Bifidobacterium, Roseburia* and *Blautia* [62]. 

The relationship between protein intake and microbiota composition, and the subsequent consequences for the host metabolism, are thus very complex and not fully understood. The effects of protein intake on the gut microbiota composition, and the consequences for muscle mass deposition, may depend on protein quality and microbiota metabotype [54], so that the pro-anabolic response to protein intake, mediated by the microbiota, may be different on an individual basis. 

### 2.2. Microbiota in Muscle-Wasting Disorders

Very few studies have directly assessed the possible association between gut microbiota composition and muscle mass, or the effects of microbiota manipulations on muscle mass, and all of them have been performed in animal models. Siddhart et al. [63] have demonstrated that rats with age-related sarcopenia exhibit a distinct gut microbiota composition compared to rats with a normal muscle mass. The sarcopenic rat microbiota also showed different functionalities, with reduced representation of genes involved in carbohydrate, protein, lipid digestion, and vitamin biosynthesis [63]. The reduced bioavailability of macronutrients and vitamins possibly associated with these age-related functional changes may play a relevant pathophysiological role in determining a reduction of muscle mass, as hypothesized by some authors [21,22]. 

Additionally, the presence of muscle atrophy in ghrelin-null mice is associated with a pro-inflammatory composition of gut microbiota, with selective depletion of butyrate-producing bacteria such as *Clostridium XIVa* and *Roseburia* [64]. Mice infected with *Toxoplasma gondii* exhibit a profound gut microbiota dysbiosis, that is associated with pro-inflammatory status and muscle wasting leading to cachexia, although a similar microbiota composition in uninfected co-housing mice was not associated with cachexia [65].

The administration of *Lactobacillus reuteri* to mouse models of cachexia resulted in increased muscle weight and muscle fiber size, an effect probably mediated by the up-regulation of FoxN1 transcription factor resulting in reduced systemic inflammation [66]. In a mouse model of leukemia, the administration of a probiotic blend containing several Lactobacilli was associated with reduced inflammation and reduced levels of atrogin-1 and other muscle atrophy markers [67]. In another study, the administration of the SCFA producer *Faecalibacterium prausnitzii* to mice fed with high-fat diet resulted in increased grastrocnemius muscle mass, and increased expression of mitochondrial respiratory chain complexes [68]. However, the capacity of *Faecalibacterium prausnitzii* to produce SCFA is mediated by interactions with other microbial species, including Bifidobacteria [69,70]. Since the abundance of Bifidobacteria is negatively influenced by high-protein diets, the results of this study should be interpreted with caution and need future confirmation under different dietary regimens. Finally, the administration of butyrate, a SCFA produced by gut microbiota and known for its anti-inflammatory and pro-anabolic effects due also to the capacity of inhibiting the enzyme histone deacetylase, to aged mice resulted in improved muscle lean mass and cross-sectional area [71].

These studies underline the role of gut microbiota-derived metabolites in the promotion of skeletal muscle anabolism. These metabolites may not include only SCFAs, but also several phenolic compounds and their conjugates. A recent in vitro study has shown that phenolic compounds produced by the microbiota can increase glucose uptake in muscle fibers, inducing anabolic responses that increase muscle mass [72]. The mitochondria, whose pathophysiological role in the onset of sarcopenia is well established [73], may be also involved. 

**Figure 1:**
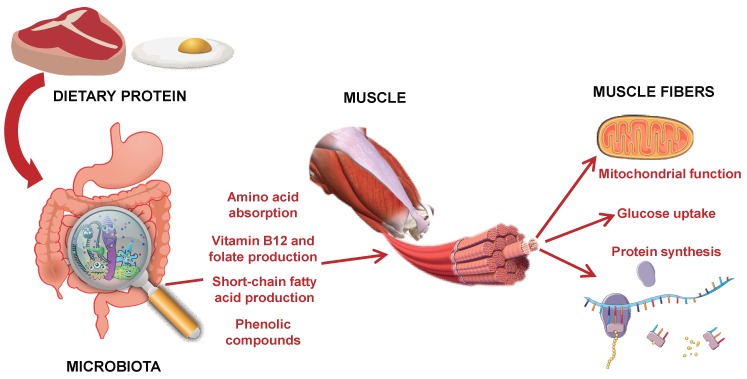
Overview of the main putative mechanisms supporting a role of gut microbiota in modulating nutrient signals to the skeletal muscle.

The possible mechanisms linking diet, microbiota composition and skeletal muscle mass synthesis are summarized in Figure 1. In the current literature, there is however no direct evidence of an association between gut microbiota composition and skeletal muscle mass in human beings, and particularly in the context of age-related sarcopenia [48]. 

Although the possibility that the intestinal microbiota represents an active modulator of muscle mass deposition is intriguing from a pathophysiological and clinical point of view, the circumstance that microbes develop symbiotic relationship with the host should also be considered. Therefore, sarcopenia-associated changes in gut microbiota composition and functionality, as those observed in animal studies [63,64,65], may simply reflect the consequence of a reduced metabolic capacity of the host, or the consequence of sarcopenia itself. This hypothesis has not been verified either in animal or human studies. However, reduced physical activity and exercise, representing a common feature of sarcopenic subjects, are independently associated with unfavorable changes in microbiota composition [74], suggesting, as discussed further, a bidirectional cross-talk between muscle and gut microbiota. 

## 3. Gut Microbiota and Parameters of Muscle Function

### 3.1. Gut Microbiota and Muscle Strength

Reduction in muscle strength is an important characteristic of sarcopenia and physical frailty, concurring to determine the age-related decline in physical performance [75,76]. For clinical purposes, muscle strength is evaluated by measuring the hand-grip strength of the dominant side with a hand-held dynamometer [77]. This parameter physiologically declines with aging, but its reductions are generally more pronounced in those who fulfil criteria for sarcopenia and physical frailty [78,79]. 

Very few studies centered on human gut microbiota have considered the possible correlation with hand-grip strength. In a recent observational study, Bjørkhaugh and colleagues compared the fecal microbiota composition of a group of 24 subjects with alcohol overconsumption and 18 controls, considering nutritional status and hand-grip strength as clinical metadata [80]. They found that alcohol overconsumers had lower hand-grip strength, higher relative abundance of Proteobacteria, *Sutterella, Clostridium* and *Holdemania* and lower relative abundance of *Faecalibacterium*. These alterations in fecal microbiota composition were also accompanied by reduced fecal levels of SCFAs, indicating a pro-inflammatory microenvironment [80]. Although the alterations of gut microbiota were probably determined by alcohol consumption and suboptimal nutritional status, these results suggest that the microbiota could have some influence on muscle strength. 

In a randomized controlled trial testing the effect of a prebiotic containing inulin and fructooligosaccharides vs placebo in 60 nursing home residents, the intervention group experienced a significant improvement in hand-grip strength (12.4 ± 3.2 vs. 10.2 ± 4.1 Kg, p = 0.04) after 13 weeks [81]. Although the fecal microbiota composition was not assessed in this study, inulin and fructooligosaccharides have known beneficial effects on gut microbiota composition [82,83,84]. Inulin supplementation can in fact improve the relative abundance of *Bifidobacterium, Anaerostipes* and *Bilophila* in human feces [82], while fructooligosaccharides have the capacity of selectively increasing the abundance of bifidobacteria [83,84]. Prebiotics also stimulate the microbial synthesis of SCFAs, that can improve calcium absorption and bone mineralization [85]. Bone health and calcium homeostasis are critically associated with the onset of sarcopenia and physical frailty, as suggested by a recent systematic review [86]. Thus, one can hypothesize that the observed benefices on hand-grip strength may be mediated by specific changes in gut microbiota composition, modulating inflammation and anabolic balance. 

However, recent studies conducted on mouse models have put this hypothesis into question. The administration of a high-protein beef extract improved grip strength in both mice harboring a physiological gut microbiota and germ-free mice, indicating that the effect of dietary proteins on muscle strength is not mediated by gut microbial communities [87]. In another experimental study, the administration of resveratrol, a polyphenol compound able to promote skeletal and cardiac muscle cell biogenesis and remodeling, to mouse models of heart failure resulted in significant changes in intestinal microbiota composition, but no improvement in muscle strength [88]. These results do however need confirmation in human studies before they can be considered valid for clinical application. 

In summary, an association between human gut microbiota composition and muscle strength is probable, with *Bifidobacterium* and *Faecalibacterium* as the most important putative players. The existence of a cross-feeding interaction between these two genera, so that the capacity of butyrate synthesis by *Faecalibacterium* is mediated by the abundance of Bifidobacteria [70], should be also considered in the possible muscle strength-microbiota association. However, more studies are needed before we can conclude that the microbiota is a clinically significant determinant of muscle strength in aging. 

### 3.2. Gut Microbiota and Gait Speed

The walking speed is considered a fundamental parameter related to the health status of older patients [77,89]. In fact, it is able to predict the onset of mobility-disability and mortality, and correlates with the clinical course of several chronic illnesses [90]. The association of slow gait speed (≤0.8 m/s) with incident disability has been particularly emphasized, although the cut-offs may be different in different settings and populations [91]. Recent studies also suggest that the association between gait speed and health outcomes is mediated by the habitual level of physical activity [92]. 

Gait speed is generally measured on a 4-m linear path, recommending the patient to walk at the usual speed [77]. The use of electronic devices for measurement is particularly recommended, improving precision and repeatability of measures [93]. 

Few studies have assessed the relationship between gut microbiota composition and gait speed. In an experimental study conducted on the fruit fly *Drosophila melanogaster*, the presence of germ-free status or the administration of antibiotics resulted in hyperactive locomotor behavior, with faster locomotion than animals with physiological microbial colonization in the gut [94]. The administration of specific bacteria, including *Lactobacillus*, to germ-free animals restored normal locomotor behavior [94]. These results suggest that the intestinal microbiota could influence locomotion by modulating sugar metabolism and activation of octopaminergic system (the equivalent to noradrenergic system in humans). However, the intestinal microbiota and the physiology of *Drosophila melanogaster* are much different than the human ones (5–20 microbial species vs more than 1000 harbored in the human gut), so the translation of these findings to humans needs further investigation. 

The human studies investigating the association between gut microbiota composition and gait speed are summarized in Table 1. In older subjects with Parkinson’s disease, the relative abundance of some taxa were significantly correlated with gait speed and postural instability [95,96]. Namely, the relative abundance of Enterobacteriaceae was found as positively associated with gait difficulty and postural instability in 72 patients with typical parkinsonism [95], while decreased Lachnospiraceae and increased Lactobacillaceae and Christensenellaceae were associated with a worse clinical profile and gait disturbances in a group of 193 patients with Parkinson’s disease, of whom 39 not under drug treatment at the time of sampling [96]. 

In a non-randomized comparative trial testing trunk muscle training vs aerobic exercise training in 32 sedentary women aged 65 or older, specific modifications in the gut microbiota composition could be detected after training, and the abundance of *Bacteroides* was positively correlated with increased gait speed at the 6-min walking distance test [97]. The *Bacteroides* abundance was correlated with cardiorespiratory fitness in another study conducted on a group of premenopausal healthy women, but gait speed was not directly assessed [98]. 

Finally, in a randomized controlled trial testing the effects of a multistrain probiotic vs. placebo in 36 patients with cirrhosis, Román and colleagues found that the beneficial modifications induced in gut microbiota by probiotic administration were associated with improvement in gait speed, but not with changes in fecal microbiota composition [99]. 

These studies overall suggest that gut microbiota composition may be associated with gait speed (Table 1). However, none of these studies was focused on older people with sarcopenia or physical frailty, and the methodology of gait speed assessment was not adherent to recommendations for geriatric patients [77]. Causality between microbiota and gait speed changes was not properly assessed by these studies, so that the influence of microbiota composition on locomotor function remains uncertain.

Moreover, gait speed represents a functional parameter that does not depend only on muscle strength and function, but also on central nervous system function. In aging, the microbiota is able to influence the brain physiology through multiple mechanisms, that have been recently reviewed by Ticinesi et al. [100] and Calvani et al. [101]. These mechanisms may also be important for the presence of frailty [100] and influence gait speed and functional dependence of older individuals. Namely, the capacity of gut bacteria to produce neurotransmitters, including γ-amino butyric acid, norepinephrine and dopamine, and to modulate the host production of serotonin, seems relevant for the association between microbiota composition and gait speed [102]. Thus, the associations, shown in Table 1, may not depend solely on the gut-muscle axis, but also on the microbial influences on the brain. 

### 3.3. Gut Microbiota and Other Parameters of Physical Performance

The assessment of physical performance is of paramount importance in geriatrics. In addition to gait speed measurement, other simple motoric tests are recommended. The most popular ones are chair-stand test, timed up-and-go test, and the Short-Physical Performance Battery (SPPB), combining three different tests (4-m walking speed, chair-stand test, balance evaluation) [77]. Alterations in the physical performance in each one of these tests, and particularly SPPB, is associated with disability, nursing home admission, falls and mortality [103,104,105]. SPPB test score performs particularly well in predicting incident disability, with a significantly higher accuracy than the measurement of gait speed alone [106]. SPPB also predicts cognitive disability [107], since physical and cognitive function are strictly inter-related [108]. 

Unfortunately, at the current literature state-of-the-art, no study has investigated the possible association of these clinical parameters of physical function with gut microbiota composition. The only exception was the study by Román and colleagues, mentioned above, where the administration of a probiotic blend to patients with cirrhosis improved the performance at timed up and go test [99]. This finding supports the existence of a gut-muscle axis, since probiotic administration is generally able to modulate microbiota composition. 

Similar intervention studies were performed in healthy adults, evaluating physical performance by measuring endurance capacity, which is not part of standard geriatric assessment [88,109]. Other studies focused on the effects of probiotic administration on the physical performance of athletes, but their results were conflicting [110,111]. However, experiments in animal models support the concept that modulation of the intestinal microbiota is associated with improved fitness and endurance, and reduced fatigability [112,113]. 

### 3.4. Gut Microbiota and Physical Frailty

In the existing studies centered on older subjects, the physical performance was evaluated in the context of frailty. Namely, frailty was operationalized in accordance with the deficit accumulation model, and most studies used the Rockwood Frailty Index as the preferred method of assessment, overlooking more specific measures of muscle function or performance [47,114,115,116,117,118,119,120]. The studies, summarized in Table 2, supported the existence of an association between frailty and fecal microbiota composition, though with different characteristics [47,114,115,116,117,118,119,120]. 

In large population studies conducted on both fit community-dwellers and frail nursing home residents, frailty was associated with reduced biodiversity of the fecal microbiota, that was partly dependent on different dietary patterns [114,115]. Frailty was also associated with reduced abundance of bacteria known for their anti-inflammatory properties, such as *Faecalibacterium prausnitzii*, and overexpression of other species, including *Eubacterium, Eggerthella, Ruminococcus* and *Coprobacillus* [115,116]. The representation of bacteria producing SCFAs was particularly reduced in nursing home residents with frailty, while fit nursing home residents showed a microbiota composition resembling to that of community-dwellers [47]. Nursing home residents also exhibited a different salivary microbiota composition in comparison with healthy controls, and the abundance of many salivary taxa, that can also colonize the intestinal tract, was correlated with frailty [118]. The association of physical components of frailty with gut microbiota composition was also confirmed as independent of covariates in a large population-based study of 1551 healthy individuals over the age of 40 [119]. Conversely, in older hospitalized patients, that generally exhibit extreme degrees of gut microbiota dysbiosis [121], the presence and severity of frailty was poorly associated with gut microbiota composition, that was instead mainly associated with the number and type of drugs administered for the treatment of acute and chronic illnesses [117]. 

Frailty was considered as the main clinical outcome in only one intervention study centered on the intestinal microbiota. Theou and colleagues randomized a group of 50 nursing home residents able to walk alone to receive a prebiotic blend (inulin and fructooligosaccharides) or maltodextrin as control for a 13-week period [120]. They observed a mild, but statistically significant, reduction in a 62-item frailty index in the intervention group [120], supporting the assumption that gut microbiota manipulation can actively influence the physical performance. An effect of direct stimulation by prebiotics on intestinal mechanical contractions, not mediated by the microbiota [122], should be also considered when interpreting the effects of prebiotic administration on the frailty index, which by definition considers multiple physiological aspects of the older person not involving only mobility [123]. 

The most evident limitation of these investigations is the methodology of assessment of physical performance. The Rockwood Frailty Index and Clinical Frailty Scale do not directly assess muscle function and performance, focusing on the presence and number of functional deficits, not involving only locomotion [124]. Thus, the results of the studies summarized in Table 2 do not allow to infer a direct association of gut microbiota composition with muscle performance in aging. However, frailty indexes are significantly associated with objective measures of muscle performance, particularly balance and chair-stand test [125,126]. Moreover, the only study, among those summarized in Table 2, where the Fried criteria were used to classify frailty, concluded that physical frailty is much more strongly associated with gut microbiota composition than cognitive frailty [119]. However, correlations between microbiota composition and objective measures of muscle performance were not assessed in that study [119]. 

## 4. The Gut-Muscle Axis: Causality or Epiphenomenon?

Existing studies suggest that the gut microbiota is associated with physical performance in aging, and that the association may be double-way [127]. Frailty and mobility-disability are in fact associated with different degrees of dysbiosis, while individuals with successful aging, such as centenarians, exhibit a fecal microbiota composition that matches the one of healthy adults [46,128,129]. These concepts support the existence of a gut-muscle axis [22], as also suggested by the only randomized controlled trial where a microbiome-centered intervention (the administration of a prebiotic blend) was associated with improved frailty index [120]. From this perspective, the gut microbiota may be actively involved in the physiopathology of sarcopenia and physical frailty, and could represent a reasonable therapeutic target [48,130]. 

On the other side, the cross-sectional design of most studies does not allow to infer a causality relationship between gut microbiota dysbiosis and reduction of muscle mass or performance. The gut microbiota is influenced by several environmental factors, that may contribute to shape its composition and functionality independently of pathophysiological processes [43]. Exercise training is a well-known modulator of gut microbiota composition, improving biodiversity and representation of taxa with purported health-promoting significance [26,131]. Conversely, sedentary behavior is associated with dysbiosis, overgrowth of opportunistic pathogens and with different microbiome functionality [132]. From this perspective, the observed landmarks of sarcopenia and physical frailty in the fecal microbiota may simply represent the consequence of reduced physical activity [133], just an epiphenomenon of pathophysiological processes driven by environmental factors [134]. 

The role of diet makes this relationship even more complicated. Dietary protein supplementation is generally considered one of the main therapeutic measures for sarcopenia and physical frailty [14,15], and some animal investigations seem to support a role of the microbiota in facilitating protein digestion, fermentation and promotion of muscle anabolism [63,64,65]. However, in most experimental models high-protein diets are generally associated with detrimental consequences for gut microbiome ecosystem, favoring overgrowth of Bacteroidetes at the expense of putative health-promoting taxa, including Bifidobacteria [59,135]. As previously discussed, an adequate representation of Bifidobacteria is fundamental for the production of butyrate [70], the most important anti-inflammatory and pro-anabolic mediator involved in the gut-muscle axis. Such modifications could have detrimental consequences for the gut-muscle axis, favoring muscle mass catabolism, rather than anabolism. Moreover, the protein source and nutritional quality could also be involved in microbiota modulation [135]. From this perspective, the advice to eat a normal quantity of proteins with high biological value, such as whey and dairy proteins, seems reasonable for its effect on anabolic balance and inflammation [136,137]. 

The current literature state-of-the-art does not help to solve the dilemmas related to gut-muscle axis. Future microbiome studies should thoroughly consider the demographical, environmental and clinical covariates that may influence microbiota composition, to highlight which associations have a true pathophysiological significance and which are instead spurious [138]. Moreover, longitudinal study designs should also be implemented, in order to verify the dynamic interactions of microbiota composition with health status in aging patients [43]. 

The intestinal microbiota is a very complex ecosystem, and its description in terms of biodiversity, taxonomy and relative abundance of taxa may not be sufficient for understanding its interactions with the host. Recent animal studies have shown that the host fitness is regulated by functional interactions among species, not by a single or a limited group of taxa [139], and that the metabolic byproducts of intestinal bacteria have a great relevance in prolonging healthspan [140]. The understanding of the possible pathophysiological role of gut microbiota in physical frailty and sarcopenia should carefully consider these aspects. 

## 5. Conclusions

The presence of a gut-muscle axis actively involved in the pathophysiology of physical frailty and sarcopenia is biologically plausible and is supported by a limited number of animal and human studies; however, the causal link remains uncertain. An association between human gut microbiota composition and muscle mass has not been demonstrated yet. In some studies, an association between microbiota composition and parameters of muscle function, such as strength, gait speed and timed up and go test, was demonstrated, while an association between microbiota and physical frailty, assessed in accordance with the deficit accumulation model, seems probable. The relationship between gut microbiota and physical performance in aging needs further investigation before it can reasonably influence clinical practice. 

## Figures and Tables

**Table 1:** Overview of the main human studies that investigated the association between fecal microbiota composition and gait speed.

First Author, Year	Study Design	Participants	Age	Type of Gait Assessment	Main Results	Taxa Correlated with Gait Speed
Scheperjans, 2015 [95]	Observational	72 patients with Parkinson’s disease; 72 controls	65 ± 6 (patients); 64 ± 7 (controls)	Clinical evaluation with UPDRS scale	Decreased abundance of Prevotellaceae in patients; Relative abundance of Enterobacteriaceae correlated with motor symptoms	Enterobacteriaceae
Barichella, 2019 [96]	Observational	193 patients with Parkinson’s disease; 22 patients with PSP; 22 patients with MSA; 113 controls	68 ± 10 (Parkinson); 71 ± 8 (PSP); 67 ± 7 (MSA); 66 ± 10 (controls)	Clinical evaluation with UPDRS scale	Decreased abundance of Lachnospiraceae in patients with PD; Abundance of three taxa associated with a worse clinical profile	Lachnospiraceae Lactobacillaceae Christensenellaceae
Morita, 2019 [97]	Non-randomized trial	32 sedentary women	70 ± 5	6-min walking distance test	Exercise intervention modified microbiota composition and improved 6-min walking distance test	*Bacteroides*
Román, 2019 [99]	Randomized controlled trial	36 patients with cirrhosis	65 ± 3	5-m walking test	Probiotic supplementation was associated with faster gait speed	None

UPDRS = Unified Parkinson’s Disease Rating Scale; PSP = Progressive Sopranuclear Palsy; MSA = Multisystemic Atrophy.

**Table 2:** Overview of the main human studies that investigated the association between fecal microbiota composition and physical frailty.

First Author, Year	Study Design	Participants	Age	Method of Physical Frailty Assessment	Main Results	Involved Taxa
Claesson, 2012 [114]	Cross-sectional	178 elderly (living in the community or in nursing homes)	78 ± 8	Functional dependence, Barthel index	Microbiota composition is related to frailty and place of residence	*Prevotella Ruminococcus Alistipes Oscillibacter*
Jackson, 2016 [115]	Cross-sectional	728 female twins living in the community	63 ± 8	39-item Frailty Index	Frailty is negatively associated with biodiversity and the relative abundance of a number of key taxa	*Eubacterium Eggerthella Faecalibacterium*
Maffei, 2017 [116]	Cross-sectional	85 community-dwellers	64 ± 7	34-item Frailty Index	The abundance of specific taxa is associated with biological aging and Frailty Index	*Eggerthella Ruminococcus Coprobacillus*
Ticinesi, 2017 [117]	Cross-sectional	76 patients hospitalized for acute illness	83 ± 8	Rockwood Clinical Frailty Scale	Frailty is not associated with biodiversity, but the abundance of a limited number of taxa	*Prevotella Oscillospira Porphyromonas Peptococcus Fonticella*
Haran, 2018 [47]	Prospective observational	23 nursing home residents	88 ± 6	Rockwood Clinical Frailty Scale	Patients with higher frailty scores exhibit lower representation of butyrate-producing bacteria	*Clostridium* cluster XIVa Lachnospiraceae *Ruminococcus*
Verdi, 2018 [119]	Cross-sectional	1551 community-dwellers from a twin cohort	63 ± 10	1–5 scale derived from the Fried phenotype	Frailty is associated with reduced microbiota biodiversity and abundance of 11 genuses	*Prevotella Lactobacillus Ruminococcus Blautia Odoribacter*
Ogawa, 2018 [118]	Cross-sectional	15 frail nursing home residents, 16 community-dwelling controls	84 ± 8 (frail); 87 ± 5 (controls)	Functional dependence	The salivary microbiota of frail patients has a different composition that that of controls	*Prevotella Actinomyces Veillonella*
Theou, 2019 [120]	Post-hoc analysis of a randomized controlled trial	50 nursing home residents without dementia	75 ± 7	62-item Frailty Index	Administration of a prebiotic blend (inulin+fructooligosaccharides) resulted in a mild reduction of Frailty Index	-
